# 

**Published:** 1860-01

**Authors:** 


### CONTENTS FOR JANUARY, 1860

### ORIGINAL COMMUNICATIONS.

Introductory Address on the Opening of the Medical Department of Lind University. By N. S. Davis, M. D. .... 1
Notes on Surgical Cases. By E. Andrews, M. D. .... 22
Cases of Puerperal Convulsions. By W. H. Byford, M. D. .... 25
Another Case. By the Senior Editor. .... 29
Case of Traumatic Abscess of the Liver, with Thoraco Abdominal Fistula. By H. Wardner, M. D. .... 33
Abstract of the Proceedings of the Chicago Medical Society .... 35
Extracts from the Records of the Chicago Academy of Medical Sciences .... 40

### SELECTIONS.

Surgical Cases in the Hospital at Milan .... 42
Cure of Spina Bifida by Application of Collodion .... 45
Remarks on Lycopus Virginicus, Prinos Verticillatus, and Epiphegus Virginianus. By Chas. A. Lee, M. D. .... 45
Employment of Veratria in Acute Diseases of the Chest .... 48
Employment of Tannin in Large Doses in Albuminous Anasarca .... 49
Saline Injections in Diptheritis .... 50
Syrup of Iodide Potassium in Syphilis .... 50

### BOOK AND PAMPHLET NOTICES.

Lectures on Surgical Pathology .... 51
Transactions of Illinois State Medical Society, 1859 .... 51

### CLINICAL REPORTS.

Medical Wards of the Mercy Hospital Service of Dr. N. S. Davis .... 55

### EDITORIAL.

Salutatory .... 61
Chicago College of Pharmacy .... 62
Transactions of State Society .... 63
Medical Department Lind University .... 63
Rush Medical College .... 64

